# Dose-dependent protective effects of *Tripterygium wilfordii* glycosides on joint and lung injury and gut microbiota remodeling in collagen-induced arthritis rats

**DOI:** 10.3389/fphar.2026.1783503

**Published:** 2026-04-23

**Authors:** Shangwen Qi, Qinghua Xing, Xiaohong Gong, Yutong Jiang, Zhina Zhao, Zhuohang Feng, Shipeng Jiao, Chunhui Wang, Songwei Li, Huan Li

**Affiliations:** 1 Department of Rheumatology and Immunology, First Affiliated Hospital of Henan University of Chinese Medicine, Zhengzhou, Henan, China; 2 First Clinical Medical College, Henan University of Chinese Medicine, Zhengzhou, Henan, China; 3 Huixian County Hospital of Traditional Chinese Medicine, Xinxiang, Henan, China; 4 Department of Gastroenterology, The First Affiliated Hospital of Henan University of Chinese Medicine, Zhengzhou, Henan, China; 5 Henan Provincial Hospital of Traditional Chinese Medicine (The Second Affiliated Hospital of Henan University of Traditional Chinese Medicine), Zhengzhou, Henan, China; 6 The Second Clinical Medical College of Henan University of Traditional Chinese Medicine, Zhengzhou, Henan, China

**Keywords:** fibrosis, gut microbiota, gut-lung axis, inflammation, RA-ILD, TWG

## Abstract

**Background:**

Rheumatoid arthritis-associated interstitial lung disease (RA-ILD) is a major cause of morbidity and mortality in patients with rheumatoid arthritis (RA). *Tripterygium wilfordii Hook.f. [Celastraceae]* glycosides (TWG) are used in RA treatment, but their dose-response relationship and involvement of the gut-lung axis in RA-ILD remain unclear.

**Methods:**

Collagen-induced arthritis (CIA) was established in Wistar rats. Animals were assigned to normal control (NC), model, TWG-L (9 mg/kg), TWG-M (45 mg/kg), TWG-H (90 mg/kg), and prednisolone (Pred, 5 mg/kg) groups. Treatments were administered by oral gavage from day 15 to day 42, and animals were sacrificed on day 43. Joint and lung pathology was assessed histologically. Fecal microbiota composition was analyzed by 16S rRNA gene sequencing, and microbial functional profiles were predicted using PICRUSt2. RT-qPCR was used to quantify IL-6 and TNF-α mRNA expression in joint and lung tissues, while Western blot analysis was used to detect TGF-β1 and α-SMA protein expression in lung tissue.

**Results:**

TWG ameliorated both arthritis severity and pulmonary injury in a dose-dependent manner, with the strongest effects observed in the TWG-H group, showing efficacy comparable to Pred group. Histopathological analysis showed marked improvement in synovial inflammation, joint destruction, alveolar septal thickening, inflammatory cell infiltration, and collagen deposition. RT-qPCR demonstrated significant reductions in IL-6 and TNF-α mRNA expression in joint and lung tissues, while Western blot analysis showed decreased pulmonary TGF-β1 and α-SMA expression, indicating coordinated anti-inflammatory and anti-fibrotic effects. TWG also induced dose-dependent remodeling of the gut microbiota. High-dose TWG shifted the microbial community structure toward that of the NC group, increased the relative abundance of *Lactobacillus* and several butyrate-producing genera, and reduced *Desulfovibrio*, *Bilophila*, and *Prevotella*. PICRUSt2 predicted enhanced metabolic potential for short-chain fatty acid-related pathways and reduced xenobiotic degradation in the TWG-H group.

**Conclusion:**

TWG, particularly in the high-dose group, exerted dual protective effects on joint and lung injury in CIA rats. These effects were associated with suppression of inflammatory cytokines and fibrosis-related proteins, with partial restoration of gut microbiota homeostasis. The findings support TWG as a potential anti-inflammatory and anti-fibrotic intervention for RA-ILD, although the microbiota-related effects remain associative and require further mechanistic validation.

## 1 Introduction

Rheumatoid arthritis (RA) is a chronic, systemic autoimmune inflammatory disease characterized by persistent synovitis, which leads to progressive cartilage and bone destruction and ultimately results in joint deformity and functional impairment (Aletaha et al., 2010). Interstitial lung disease (ILD) represents one of the most common and severe extra-articular manifestations of RA (Wu et al., 2025). Rheumatoid arthritis-associated interstitial lung disease (RA-ILD) is defined by radiological, pulmonary functional, or histopathological evidence of interstitial lung involvement in patients with RA (Johnson et al., 2023). Clinically apparent RA-ILD affects approximately 5%–15% of RA patients (Groseanu and Niță, 2024). Following diagnosis, the reported median survival ranges from 3 to 10 years, and RA-ILD is associated with a markedly increased risk of mortality (Juge et al., 2023).

*Tripterygium wilfordii Hook.f. [Celastraceae]* glycosides (TWG) are a group of bioactive plant metabolites extracted from the traditional Chinese medicinal botanical drug *Tripterygium wilfordii Hook.f. [Celastraceae]* (Zhao and Liu, 2022). TWG exerts immunomodulatory and antifibrotic effects in RA-ILD (Zheng et al., 2021). Used either as monotherapy or in combination with disease-modifying antirheumatic drugs (DMARDs), TWG can attenuate inflammatory activity by inhibiting key signaling pathways, including NF-κB and TGF-β (Chen et al., 2018), and by reducing the production of pro-inflammatory cytokines such as TNF-α, IL-6, IL-1β, and IL-17 (Feng et al., 2022). Through these mechanisms, TWG may contribute to the attenuation of pulmonary fibrotic progression (Mohd Khairudin et al., 2023). However, emerging evidence suggests that the therapeutic efficacy of TWG in RA-ILD is dose-dependent, with different dosing regimens potentially yielding distinct clinical outcomes (Zhang et al., 2021; Lin et al., 2020). Therefore, evaluating the dose-response relationship of TWG in RA-ILD is warranted.

The gut-lung axis has garnered increasing attention in RA-ILD research. Although previous studies have demonstrated that TWG can ameliorate pulmonary inflammation and fibrosis through multiple molecular mechanisms, evidence regarding its regulatory effects on gut microbiota homeostasis and the involvement of the gut-lung axis remains limited. In the present study, collagen-induced arthritis (CIA) was established in rats, in which spontaneous pulmonary interstitial lesions were observed. TWG was administered at different doses to investigate its dose-dependent therapeutic effects on both joint and lung injury. In addition, its anti-inflammatory and antifibrotic effects were validated at the molecular level by assessing key inflammatory and fibrosis-related markers. Furthermore, this study aimed to establish hypothesis-generating, association-based links between gut microbiota alterations and therapeutic outcomes, thereby informing dosing strategies and identifying candidate microbiome biomarkers for future validation.

## 2 Materials and methods

### 2.1 Materials

#### 2.1.1 Animals

Sixty 10-week-old female specific pathogen-free (SPF) Wistar rats, weighing 170–200 g, were obtained from Beijing Sibefu Biotechnology Co., Ltd. (License No.: SCXK (Jing) 2019-0010). All experimental procedures were approved by the Experimental Animal Ethics Committee of the Second Affiliated Hospital of Henan University of Chinese Medicine (Approval No.: AF/SC-04/04.0) and were conducted in strict accordance with relevant guidelines for the care and use of laboratory animals.

#### 2.1.2 Reagents and instruments

Complete Freund’s adjuvant (Lot: SLCL9648), incomplete Freund’s adjuvant (Lot: SLCM2985), bovine type II collagen (immunization grade; Lot: 20022), hematoxylin-eosin (H&E) staining kit, Masson’s trichrome staining kit, and paraformaldehyde were purchased from Zhengzhou Dianjing Technology Co., Ltd. TWG tablets (10 mg/tablet; National Drug Approval No.: Z33020422) were manufactured by Zhejiang Deende Pharmaceutical Co., Ltd., and prednisolone acetate tablets (5 mg/tablet; National Drug Approval No.: H33021207) were produced by Zhejiang Xianju Pharmaceutical Co., Ltd. All drugs were supplied by the First Affiliated Hospital of Henan University of Chinese Medicine.

### 2.2 Animal experiments

#### 2.2.1 Model establishment and intervention

CIA was established using bovine type II collagen emulsified with Freund’s adjuvant. Primary immunization was performed 7 days before booster immunization. Rats in the experimental groups received subcutaneous injections of collagen emulsion at the back, tail base, and right hind paw, followed by a booster injection 7 days later using collagen emulsified with incomplete Freund’s adjuvant. Rats in the normal control (NC) group received equivalent volumes of phosphate-buffered saline at the same anatomical sites. Wistar rats were selected based on prior evidence demonstrating their susceptibility to CIA (Song et al., 2015; Inglis et al., 2007; Bäckström and Dahlgren, 2008).

For time-course analyses, the day of Primary immunization was designated as day 0. Successful model establishment was confirmed by inflammatory swelling of the contralateral hind paw. Modeled rats were then randomly assigned to five groups (n = 10 per group): model group, low-dose TWG (TWG-L), medium-dose TWG (TWG-M), high-dose TWG (TWG-H), and prednisolone (Pred) group. Oral gavage was initiated on day 15 and continued once daily for 28 consecutive days (day 15–42). This 4-week treatment period allowed for the spontaneous development of pulmonary interstitial lesions (Yang et al., 2022), with terminal sample collection performed on day 43. The experimental design and timeline are shown in Figure 5A.

Dose selection for TWG was based on body surface area (BSA)-based interspecies dose conversion. The standard clinical oral dose of Tripterygium glycoside tablets is 1 mg/kg/day, with a maximum recommended dose of 1.5 mg/kg/day (Na et al., 2020). Using BSA conversion (Reagan-Shaw et al., 2008), the rat-equivalent dose corresponding to the maximum clinical dose is approximately 9 mg/kg. Accordingly, 9 mg/kg was defined as the low dose, while 45 mg/kg and 90 mg/kg were selected as medium and high doses, respectively, to establish a graded dose range for dose-response evaluation.

Prednisolone acetate (5 mg/kg) was used as a therapeutic positive control rather than an inducer of arthritis. In this study, CIA was induced by bovine type II collagen and Freund’s adjuvant, whereas prednisolone was administered only during the treatment phase after successful model establishment. Its selection was based on its well-established anti-inflammatory properties and prior use as a reference glucocorticoid in CIA models (Zimmermann et al., 2009; Gao et al., 2021). TWG and prednisolone were freshly suspended in saline before administration. Rats in the NC and model groups received equivalent volumes of saline. All animals had free access to food and water, and clinical status was monitored daily. Arthritis index (AI) scoring and paw swelling measurements were conducted at predefined time points.

#### 2.2.2 Lung coefficient

Five rats from each group were randomly selected. After blood collection via the abdominal aorta, the thoracic cavity was opened, and the entire lung was excised. Surface blood was gently rinsed with saline, and excess moisture was removed using filter paper. Lung wet weight was measured using a precision balance. The lung coefficient was calculated (Kadam and Schnitzer, 2023):

$$\text{Lung coefficient}\;\left(\%\right)=\frac{\text{Lung}\;\text{wet}\;\text{weight}\;\left(g\right)}{\text{Body}\;\text{weight}\;\left(\text{g}\right)}\times 100\%$$

#### 2.2.3 Paw thickness measurement

Five rats from each group (NC, model, TWG-L, TWG-M, TWG-H, and Pred) were randomly selected for paw thickness assessment. Measurements were performed using a calibrated digital vernier caliper (resolution: 0.01 mm) by a single operator blinded to group allocation. Paw thickness was recorded at the transverse diameter of the third proximal phalanx of the hind paw.

#### 2.2.4 Histopathological examination

Ankle joints and lung tissues were fixed in 4% paraformaldehyde, paraffin-embedded, sectioned, and stained with H&E. Lung sections were additionally stained with Masson’s trichrome for fibrosis assessment. For each animal, one section was analyzed. Two non-overlapping microscopic fields per section were selected at ×10 magnification using a fixed scale bar of 100 μm. Field selection followed a predefined rule to exclude large airways and vessels and to ensure representation of parenchymal regions; the same criteria were applied across all groups. Histopathological scoring was conducted independently by two assessors blinded to group allocation. Field-level scores were averaged within each section, and the mean of the two assessors’ scores was used as the final value for each animal. Discrepancies were resolved by consensus. Inter-rater reliability was not formally quantified.

Arthritis severity of ankle joints was graded using a semiquantitative composite score on a 0–4 ordinal scale based on synovial hyperplasia and inflammatory cell infiltration, pannus formation, cartilage damage, and bone erosion (Hayer et al., 2021). Score 0 indicated normal synovium with intact cartilage and bone and no inflammatory infiltration. Score 1 indicated mild synovial thickening with sparse inflammatory infiltration and preserved joint architecture. Score 2 indicated moderate synovial hyperplasia with increased inflammatory infiltration and early superficial cartilage irregularity or erosion. Score 3 indicated severe synovial hyperplasia with pannus formation, marked inflammatory infiltration, and evident cartilage erosion with marginal bone damage. Score 4 indicated very severe destructive arthritis with extensive pannus, profound architectural disruption, large cartilage defects or loss, and prominent bone erosion with joint space narrowing or collapse.

Pulmonary inflammation was graded using a semiquantitative ordinal scale from 0 to 4 based on alveolar septal thickening, the extent and severity of inflammatory cell infiltration, and disruption of alveolar architecture (Ren et al., 2025). Score 0 indicated no apparent inflammation with preserved alveolar structure and minimal inflammatory cells. Score 1 indicated mild focal inflammatory infiltration with mild septal thickening. Score 2 indicated mild multifocal inflammation with clear but non-diffuse infiltration and moderate septal thickening. Score 3 indicated moderate widespread infiltration with marked septal thickening and partial involvement or collapse of alveolar spaces. Score 4 indicated severe diffuse infiltration with prominent structural destruction or consolidation-like changes and extensive involvement of alveolar spaces.

Pulmonary fibrosis was evaluated on Masson’s trichrome sections using an Ashcroft-type ordinal scale from 0 to 5 as previously described (Zhou et al., 2023). Score 0 indicated no collagen deposition and a normal alveolar structure. Score 1 indicated minimal focal collagen deposition with mild septal thickening. Score 2 indicated mild fibrosis with increased collagen deposition and thickened septa affecting limited areas. Score 3 indicated moderate fibrosis with broader interstitial collagen accumulation and partial distortion of alveolar architecture. Score 4 indicated severe fibrosis with extensive collagen deposition and marked architectural distortion. Score 5 indicated very severe fibrosis with widespread collagen deposition and near-complete loss of normal alveolar structure.

#### 2.2.5 AI scoring

Arthritis severity was evaluated using a standardized 5-point scoring system for each hind paw: 0: normal paw with no redness or swelling; 1: mild redness or swelling of the interphalangeal joints; 2: moderate redness or swelling of the interphalangeal and metatarsophalangeal joints; 3: marked redness and swelling involving the entire paw below the ankle; 4: severe swelling accompanied by joint rigidity and functional impairment. Scores from both hind paws were summed to generate a total AI score of 0–8 (Du et al., 2022). A total score ≥4 indicated successful model establishment.

#### 2.2.6 16S rRNA sequencing and analysis

Gut microbiota profiling was performed using a standard high-throughput 16S rRNA gene sequencing workflow. Total genomic DNA was extracted from fecal samples, quantified using a NanoDrop spectrophotometer, and assessed for integrity by 1.2% agarose gel electrophoresis. The V3-V4 hypervariable regions of the bacterial 16S rRNA gene were amplified using barcoded universal primers and TransStart Pfu high-fidelity DNA polymerase. PCR amplicons were purified using VAHTS™ DNA Clean Beads (0.8× ratio), quantified via the PicoGreen assay, and pooled at equimolar concentrations. Sequencing libraries were constructed using the Illumina TruSeq Nano DNA LT Kit, including end repair, A-tailing, adapter ligation, PCR enrichment, and size selection (2% agarose gel). Libraries with concentrations ≥2 nM were sequenced on the Illumina MiSeq PE300 platform (insert size: 200–450 bp).

Raw reads were processed using the QIIME2 pipeline with DADA2 for quality control, denoising, and amplicon sequence variant (ASV) inference. Downstream analyses included: (i) taxonomic composition profiling; (ii) alpha diversity analysis (Chao1 and Shannon indices) with rarefaction curves; (iii) beta diversity analysis using principal coordinate analysis (PCoA) and PERMANOVA; (iv) differential taxonomic biomarker identification using LEfSe; (v) microbial co-occurrence network analysis; and (vi) functional prediction using PICRUSt2.

#### 2.2.7 RT-qPCR validation of inflammatory markers

Five animals per group were randomly selected, and lung and joint tissues were collected for RT-qPCR analysis. Approximately 20 mg of tissue was homogenized in TRIzol reagent with zirconia beads under cold conditions. Total RNA was extracted according to the manufacturer’s protocol, and RNA concentration and purity were measured using a NanoDrop 2000 spectrophotometer. Equal amounts of RNA (2 μg) were reverse-transcribed in a 20 μL reaction system using the ReverTra Ace qPCR RT Kit. Quantitative PCR was performed using SYBR Green Real-Time PCR Master Mix on a TL-988 system. Each sample was analyzed in triplicate. The amplification protocol included initial denaturation at 95 °C for 30 s, followed by 40 cycles of 95 °C for 15 s and 60 °C for 30 s, with a subsequent melting-curve analysis (65 °C–95 °C). GAPDH served as the internal control, and relative mRNA expression levels were calculated using the 2^−ΔΔCt method. Primer sequences were as follows: IL-6 forward, GAC​TTC​CAG​CCA​GTT​GCC​TT; IL-6 reverse, AAG​TCT​CCT​CTC​CGG​ACT​TGT; TNF-α forward, CGT​CAG​CCG​ATT​TGC​CAT​TT; TNF-α reverse, TCC​CTC​AGG​GGT​GTC​CTT​AG; GAPDH forward, ACA​GCA​ACA​GGG​TGG​TGG​AC; GAPDH reverse, TTT​GAG​GGT​GCA​GCG​AAC​TT.

#### 2.2.8 Western blot (WB) validation of fibrotic markers

Lung tissues were lysed in RIPA buffer supplemented with PMSF, homogenized on ice, and centrifuged at 10,000–14,000 × g for 3–5 min to obtain supernatants. Protein concentrations were determined using a BCA assay kit. Equal amounts of protein were mixed with loading buffer, denatured, separated by SDS-PAGE, and transferred onto PVDF membranes. After blocking, membranes were incubated overnight at 4 °C with primary antibodies against α-SMA, TGF-β1, and GAPDH, followed by incubation with HRP-conjugated secondary antibodies at room temperature for 30 min. Protein bands were visualized via enhanced chemiluminescence (ECL) and quantified with Image-Pro Plus 6.0. Relative protein expression was calculated as the ratio of target protein intensity to GAPDH.

#### 2.2.9 Statistical analysis

Statistical analyses were performed using GraphPad Prism 10.0 and R version 4.2.2. A two-sided p < 0.05 was considered statistically significant. Data are presented as mean ± standard deviation (SD) for approximately normally distributed variables, and as median with interquartile range (IQR) for non-normally distributed or ordinal data. For multiple-group comparisons, one-way ANOVA was applied to parametric data. Nonparametric or ordinal data were analyzed using the Kruskal-Wallis test, followed by Dunn’s *post hoc* test with Holm adjustment for multiple comparisons versus the model group.

Histopathological sections were scored independently by two blinded assessors. The mean of the two scores for each animal was used for statistical analysis.

For microbiome analyses, alpha diversity indices were compared using the Kruskal-Wallis test. Beta diversity was assessed using PERMANOVA with 999 permutations. Differential abundance analysis was performed using metagenomeSeq with false discovery rate (FDR) control (FDR<0.05). PICRUSt2 results were interpreted as predicted metabolic potential rather than direct measurements of functional activity.

## 3 Results

### 3.1 Identification of the RA rat model and evaluation of therapeutic effects

**FIGURE 1:**
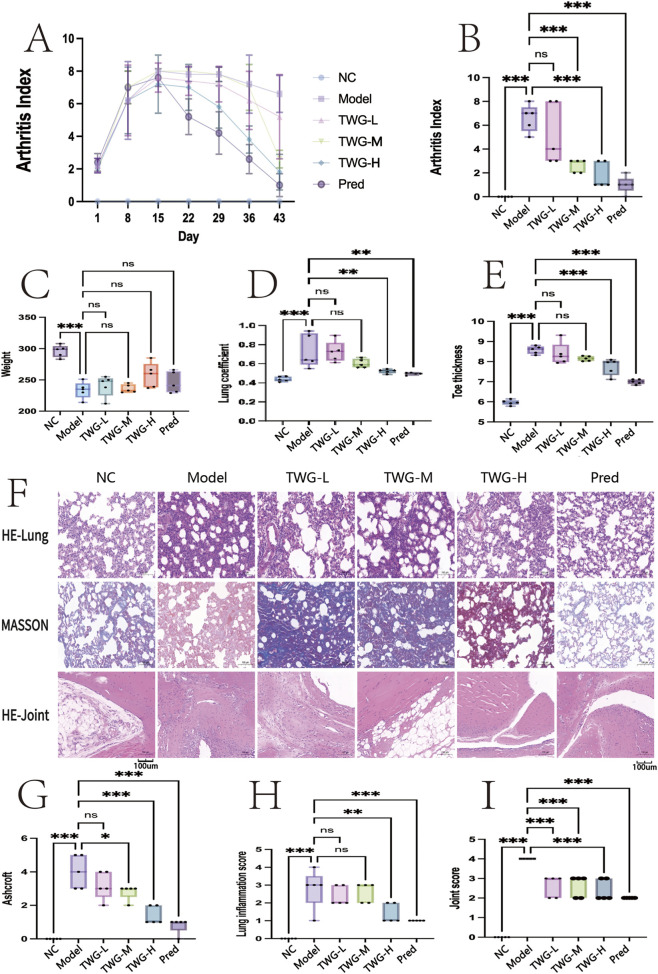
Clinical phenotypes and histopathological changes in CIA-induced RA-ILD rats and the therapeutic effects of TWG. Groups: NC; model; TWG-L, low-dose TWG; TWG-M, medium-dose TWG; TWG-H, high-dose TWG; Pred, prednisolone (positive control). **(A)** Time course of the arthritis index (AI) during the experimental period. (mean ± SD, n = 10 per group) **(B)** AI at the endpoint (day 43). **(C)** Body weight at the endpoint. **(D)** Lung coefficient at the endpoint. **(E)** Toe (paw) thickness at the endpoint. **(F)** Representative histological images: H&E staining of lung tissue (top), Masson’s trichrome staining of lung tissue (middle), and H&E staining of ankle joint tissue (bottom), showing inflammatory cell infiltration, tissue architectural disruption, collagen deposition, and synovial hyperplasia across groups. **(G)** Pulmonary fibrosis quantified on Masson’s trichrome-stained lung sections using the (modified) Ashcroft score. **(H)** Lung inflammation score quantified on H&E-stained lung sections. **(I)** Joint histopathology score quantified on H&E-stained ankle joint sections. Data are presented as individual animals (n = 5 per group) and summarized as mean ± SD for bar graphs **(B–E)**, or as box-and-whisker plots (median with interquartile range (IQR)) with individual dots for nonparametric outcomes **(G–I)**. Overall group differences were assessed using the Kruskal-Wallis test. Pre-specified pairwise comparisons versus the Model group were performed using Mann-Whitney U tests with Holm adjustment for multiple testing **(B)**, whereas multiple comparisons across groups for histological scores were conducted using Dunn’s *post hoc* test **(G–I)**. ns, not significant; p < 0.05, p < 0.01, p < 0.001. Abbreviations: CIA, collagen-induced arthritis; RA-ILD, rheumatoid arthritis-associated interstitial lung disease; TWG, *Tripterygium wilfordii Hook.f. [Celastraceae]* glycosides; H&E, hematoxylin and eosin; AI, arthritis index; SD, standard deviation.

Successful establishment of the RA model was confirmed, and therapeutic efficacy was subsequently evaluated across groups. In the model group, AI increased rapidly following booster immunization (day 0), peaked between days 15 and 22, and gradually declined thereafter, remaining elevated through day 43 (Figure 1A). In contrast, the NC group maintained an AI score of 0 throughout the study. Early AI trajectories in all treatment groups paralleled those in the model group, indicating comparable arthritis induction across groups.

From day 15 onward, AI declined markedly in the Pred group, reaching the lowest levels by day 43. The TWG-H group showed a sustained reduction after day 22, whereas the TWG-M group exhibited a more gradual decline, and improvement was least pronounced in the TWG-L group. At day 43, AI differed significantly among groups (Kruskal-Wallis test: H = 24.89, p = 0.00015). In pre-specified pairwise comparisons versus the model group (Mann-Whitney U test with Holm adjustment; m = 5), AI scores were significantly lower in the Pred, TWG-H, and TWG-M groups (adjusted p = 0.0426 for each), whereas the TWG-L group did not reach statistical significance (adjusted p = 0.5232; Figure 1B). Exploratory comparison between Pred and TWG-H showed no significant difference (p = 0.2877). These findings confirm robust model induction and demonstrate a dose-dependent trend toward attenuation of arthritis severity with the *Tripterygium wilfordii* botanical drug. Greater numerical efficacy was noted at higher doses.

### 3.2 Evaluation of paw thickness and lung coefficient

Body weight differed significantly among groups (Kruskal-Wallis test: H(5) = 15.45, p = 0.0086; Supplementary Table S1), with the highest values observed in the NC group and the lowest in the model group. In pre-specified comparisons versus the model group (Mann-Whitney U test with Holm adjustment; m = 5), only the NC group showed significantly higher body weight (p_Holm = 0.0397), whereas differences were insignificant for TWG-L, TWG-M, TWG-H, or Pred groups (Figure 1C; Supplementary Table S1).

The lung coefficient also differed significantly among groups (H(5) = 25.41, p = 1.16 × 10^−4^; Figure 1D; Supplementary Table S1). Compared with the model group, lung coefficients were significantly reduced in the NC, TWG-H, and Pred groups (p_Holm = 0.0397 for each), whereas no significant differences were observed in the TWG-L or TWG-M groups.

Similarly, paw thickness showed significant intergroup differences (H(5) = 25.04, p = 1.37 × 10^−4^; Figure 1E). Relative to the model group, paw thickness was significantly reduced in the NC, TWG-M, TWG-H, and Pred groups (p_Holm = 0.0397 for each), whereas the TWG-L group did not reach statistical significance (p_Holm = 0.222). Overall, paw thickness was highest in the model group and lowest in the NC group.

### 3.3 Effect of TWG on pulmonary pathology and collagen deposition

H&E staining demonstrated preserved alveolar architecture, thin septa, and minimal inflammatory infiltration in the NC group. In contrast, the model group exhibited diffuse septal thickening, prominent inflammatory infiltration, and alveolar exudation. TWG treatment reduced inflammatory cell infiltration and septal thickening in a dose-dependent manner, with the TWG-H group showing only mild residual inflammation, comparable to the Pred group (Figures 1F,H). Pulmonary inflammation scores differed significantly among groups (Kruskal-Wallis test: H = 23.58, p = 0.000262). In planned comparisons versus the model group (Dunn’s test with Holm correction; m = 5), only the NC group remained significantly lower (p_Holm = 0.00074). Although reductions were observed in the Pred (p_Holm = 0.1067) and TWG-H groups (p_Holm = 0.0657), these did not retain statistical significance after correction; TWG-M and TWG-L were not significant (both p_Holm = 1.00) (Supplementary Table S1).

Masson’s trichrome staining revealed minimal collagen deposition in the NC group, whereas the model group showed extensive interstitial and perivascular collagen accumulation. TWG treatment reduced collagen deposition in a dose-dependent manner, with the TWG-H group exhibiting only limited residual fibrosis, comparable to the Pred group (Figures 1F,G). Ashcroft fibrosis scores differed significantly among groups (H = 25.51, p = 0.000111). In planned comparisons, NC (p_Holm = 0.000394), Pred (p_Holm = 0.00738), and TWG-H (p_Holm = 0.0137) groups showed significantly lower fibrosis scores compared with the model group, whereas TWG-M and TWG-L were not significant after correction (both p_Holm = 0.652) (Supplementary Table S1).

### 3.4 Histopathological examination of ankle joints

H&E staining of ankle joints in the NC group revealed a thin synovial lining, smooth cartilage surfaces, and well-organized trabecular bone. In contrast, the model group exhibited marked synovial hyperplasia, vascular congestion, dense inflammatory infiltration, fibrovascular pannus formation invading cartilage, irregular joint surfaces, and focal cartilage defects. TWG treatment alleviated synovial hyperplasia and inflammatory infiltration to varying degrees, with the most pronounced improvement observed in the TWG-H group. The Pred group demonstrated near-normal joint architecture with minimal inflammation (Figures 1F,I). Semi-quantitative joint histopathology scores differed significantly among groups (Kruskal-Wallis test: H = 23.89, p = 0.000228). In planned comparisons versus the model group (Dunn’s test with Holm correction; m = 5), only the Pred group showed a significant reduction (p_Holm = 0.00752), whereas TWG-H showed a numerical decrease that did not remain significant after correction (p_Holm = 0.0556). TWG-M and TWG-L were not significant after correction (both p_Holm = 0.0958). The NC group remained significantly lower than the model group (p_Holm = 0.0000124) (Supplementary Table S1).

### 3.5 Gut microbiota diversity analysis via 16S rRNA sequencing

High-quality 16S rRNA sequencing data were obtained for all samples, with read counts at each processing stage summarized in Supplementary Table S2.

#### 3.5.1 Taxonomic composition

**FIGURE 2:**
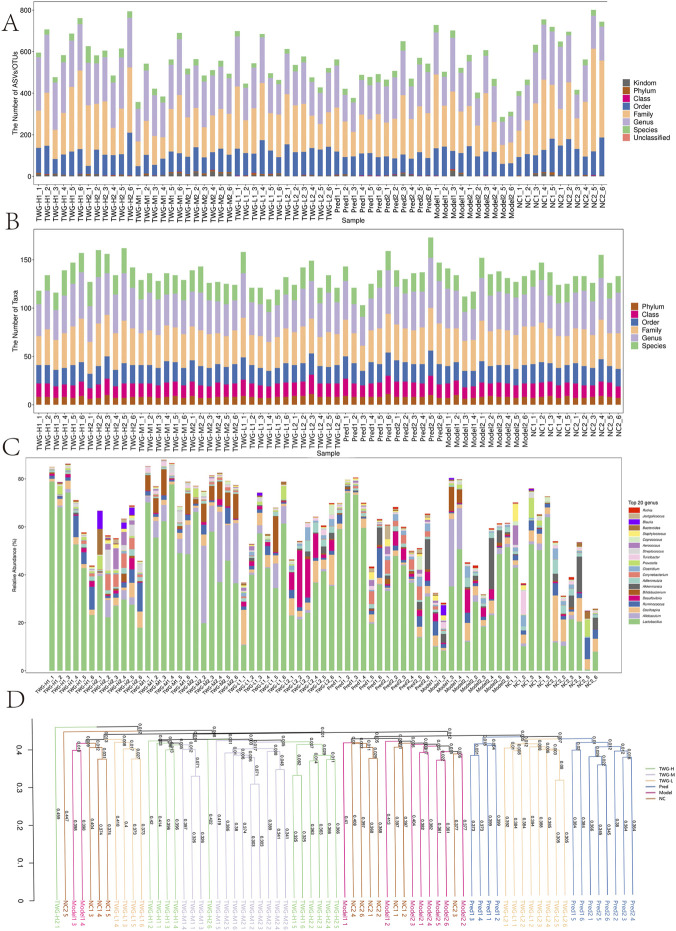
Taxonomic annotation, richness, community composition, and hierarchical clustering of gut microbiota across groups. Groups: NC; model; TWG-L, low-dose TWG; TWG-M, medium-dose TWG; TWG-H, high-dose TWG; Pred, prednisolone (positive control). **(A)** Taxonomic annotation profile per sample across major ranks (from kingdom to species), showing that most features are annotated at family/genus levels. **(B)** Numbers of annotated taxa at each taxonomic rank across samples/groups, reflecting overall classification richness. **(C)** Relative abundance of the top 20 genera across samples/groups. **(D)** Hierarchical clustering based on community dissimilarity, showing group-level separation and restorative clustering trends after TWG intervention. Abbreviations: TWG, *Tripterygium wilfordii Hook.f. [Celastraceae]* glycosides.

Across samples, approximately 300–800 ASVs were identified. Most ASVs were annotated at the family and genus levels, whereas species-level annotations were less frequent; the proportion of unassigned features was low (Figure 2A). The model group exhibited reduced total ASV counts and fewer genus-level annotations. TWG treatment increased both metrics in a dose-dependent manner, with the most pronounced improvement observed in the TWG-H group. The NC and Pred groups maintained relatively higher annotation richness across taxonomic levels (Figure 2A). The numbers of annotated genera and species per sample are summarized in Figure 2B and Supplementary Table S3. Genus-level annotations increased with escalating TWG dose, whereas improvements in species-level assignment were most evident in the TWG-M and TWG-H groups.

Community structure analysis revealed significant intergroup differences in several dominant bacterial orders, including *Lactobacillales* (H(5) = 31.45, p < 0.001), *Clostridiales* (H(5) = 17.73, p < 0.01), *Bacteroidales* (H(5) = 16.36, p < 0.01), and *Erysipelotrichales* (H(5) = 13.21, p < 0.05) (Figure 2C; Supplementary Table S4). At the genus level, *Lactobacillus* abundance was higher in the TWG-H, TWG-M, and Pred groups than in the model group. *Desulfovibrio* abundance was reduced in the TWG-H, TWG-M, and Pred groups but elevated in the TWG-L group. *Akkermansia* abundance was generally lower in all treatment groups relative to the model group. *Blautia* showed a mild increase in the TWG-treated groups, whereas *Streptococcus* increased primarily in the TWG-M group. *Allobaculum* abundance was highest in the model group, except for the TWG-M group.

#### 3.5.2 Diversity analysis

**FIGURE 3:**
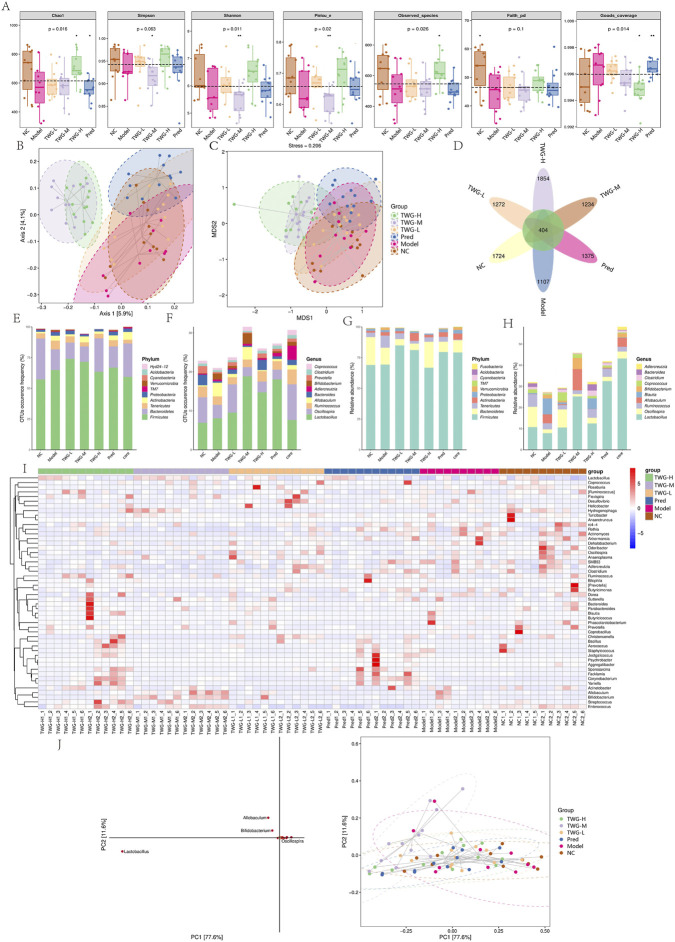
Gut microbiota diversity, taxonomic composition, and multivariate analyses across groups. Groups: NC; model; TWG-L, low-dose TWG; TWG-M, medium-dose TWG; TWG-H, high-dose TWG; Pred, prednisolone (positive control). **(A)** Alpha-diversity indices (Chao1, Simpson, Shannon, Pielou_e, Observed_species, Faith_pd, Goods_coverage) across groups. **(B)** PCoA showing beta-diversity differences among groups. **(C)** NMDS ordination of beta diversity (stress value shown in the plot). **(D)** Venn diagram showing shared and unique ASVs among groups. **(E–F)** Relative abundance profiles of major phyla **(E)** and genera **(F)**. **(G–H)** Supplementary relative abundance distributions at phylum **(G)** and genus **(H)** levels. **(I)** Genus-level heatmap with hierarchical clustering. **(J)** PCA loading plot (left) and PCA score plot (right) highlighting key genera contributing to community variation. Abbreviations: ASV, amplicon sequence variant; PCoA, principal coordinate analysis; NMDS, non-metric multidimensional scaling; PCA, principal component analysis.

Alpha diversity indices differed significantly among groups (Figure 3A; Supplementary Table S5), including Chao1 (p = 0.016), observed features (p = 0.026), Shannon index (p = 0.011), and Pielou’s evenness (p = 0.020). Faith’s phylogenetic diversity did not differ significantly (p = 0.100), while the Simpson index showed a statistically significant intergroup difference (p = 0.014).

Beta diversity analyses revealed distinct group-level separation in microbial community structure. Weighted UniFrac-based PCoA showed that the TWG-H and TWG-M groups clustered tightly and were clearly separated from other groups. The TWG-L and Pred groups partially overlapped with the NC cluster, whereas the model and NC groups overlapped in the lower-right quadrant (Figure 3B). Non-metric multidimensional scaling (NMDS) yielded a broadly consistent pattern (stress = 0.206) (Figure 3C). UPGMA clustering further demonstrated that the TWG-H and TWG-M groups diverged earlier from the main model/NC cluster, with the TWG-L and Pred groups occupying intermediate positions (Figure 2D).

#### 3.5.3 Differential abundance and biomarkers

A Venn (flower) diagram identified 404 core ASVs shared across all groups. Group-specific ASVs were most abundant in the TWG-H group (1,854), followed by NC (1,724), Pred (1,375), TWG-L (1,272), TWG-M (1,234), and the model group (1,107) (Figure 3D). At the phylum level, Firmicutes and Bacteroidetes predominated across all groups, followed by Tenericutes, Actinobacteria, and Proteobacteria, while Verrucomicrobia, TM7, Cyanobacteria, and Acidobacteria were less abundant (Figure 3E).

At the genus level, *Lactobacillus* was the dominant taxon. Its abundance was markedly reduced in the model group but increased in treatment groups, with the most pronounced restoration observed in the TWG-H and TWG-M groups (Figures 3F–H). Consistently, the relative abundance of Firmicutes declined in the model group and shifted toward NC levels following *Tripterygium wilfordii Hook.f. [Celastraceae]* treatment, particularly at higher doses, whereas the Pred group remained similar to the NC group (Figures 3G,H).

Hierarchical clustering based on genus-level abundance demonstrated clear group separation, with TWG-H and TWG-M clustering together and distinct from the TWG-L and model groups, while the Pred group occupied an intermediate position (Figure 3I). Principal component analysis (PCA) revealed a strong gradient along PC1 (77.6% of variance), clearly separating TWG-H and TWG-M from TWG-L, Pred, and NC groups. *Lactobacillus* was identified as a major contributor to PC1 loading (Figure 3J).

Differential abundance analysis using MetagenomeSeq identified significantly altered genera across 15 pairwise comparisons (FDR<0.05). Relative to the model group (and also compared with the TWG-L and Pred groups), the TWG-H group showed significant enrichment of *Lactobacillus*, *Oscillospira*, *Ruminococcus*, *Coprococcus*, *Roseburia*, and *Bifidobacterium*, alongside depletion of *Bilophila*, *Desulfovibrio*, *Prevotella*, *Aerococcus*, *Hydrogenophaga*, *Streptococcus*, and *Staphylococcus* (Supplementary Figure S1). Notably, *Allobaculum* was not enriched in the TWG-H group, being lower than or not significantly different from the model group. The TWG-M group exhibited changes in the same direction as the TWG-H group, although with smaller effect sizes relative to the model and TWG-L groups. Direct comparison between TWG-H and TWG-M indicated further enrichment of beneficial genera and further depletion of detrimental genera in the TWG-H group, supporting a dose-dependent microbiota modulation. In contrast, the Pred group displayed alterations in only a limited subset of genera compared with the model and NC groups, with partial normalization of *Lactobacillus* and *Bilophila*. The TWG-L group, when compared with the NC group, showed enrichment of *Prevotella*, *Bilophila*, and *Desulfovibrio*, while *Lactobacillus* abundance remained low (Supplementary Figure S1).

**FIGURE 4:**
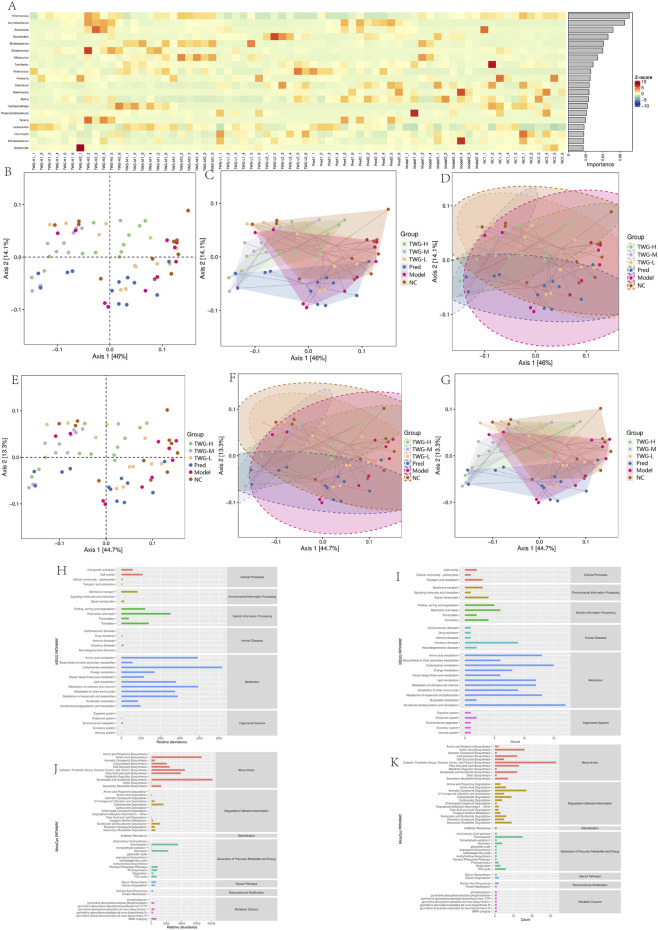
Machine learning-based identification of key genera and multivariate analyses of predicted functional profiles. Groups: NC; model; TWG-L, low-dose TWG; TWG-M, medium-dose TWG; TWG-H, high-dose TWG; Pred, prednisolone (positive control). **(A)** Z-score heatmap of genus-level relative abundance with random forest feature-importance ranking, highlighting genera contributing to group discrimination. **(B–D)** PCoA of predicted enzyme commission (EC) functional profiles: ordination plot **(B)**, group-wise convex hull visualization **(C)**, and 95% confidence ellipses **(D)**. **(E–G)** PCoA of predicted KEGG Orthology (KO) functional profiles: ordination plot **(E)**, 95% confidence ellipses **(F)**, and group-wise convex hull visualization **(G)**. **(H,I)** KEGG functional prediction: relative abundance of KEGG level-2 pathways **(H)** and differential pathways across groups **(I)**. **(J,K)** MetaCyc functional prediction: relative abundance of MetaCyc pathways **(J)** and pathway counts/frequency summary **(K)**. Abbreviations: EC, enzyme commission; KO, KEGG Orthology; PCoA, principal coordinate analysis; KEGG, Kyoto Encyclopedia of Genes and Genomes; TWG, *Tripterygium wilfordii Hook.f. [Celastraceae]* glycosides.

Random forest analysis did not identify *Allobaculum* as a positive dose-associated feature. Instead, key positive markers included *Lactobacillus*, *Oscillospira*, *Ruminococcus*, *Coprococcus*, *Roseburia*, and *Bifidobacterium*, whereas negative markers included *Bilophila*, *Desulfovibrio*, *Prevotella*, *Aerococcus*, *Hydrogenophaga*, and *Streptococcus* (Figure 4A).

#### 3.5.4 Functional PCoA analysis

To assess predicted microbial functional potential, PICRUSt2-inferred Enzyme Commission (EC) and KEGG Orthology (KO) profiles were analyzed by PCoA. For EC-level predictions, PC1 and PC2 explained 46.0% and 14.1% of the variance, respectively. The TWG-H and TWG-M groups were clearly separated from the tightly clustered model and NC groups, although partial overlap remained. Confidence ellipse analysis (95%) indicated tighter clustering in the TWG-H and TWG-M groups, while TWG-L and Pred groups occupied intermediate positions (Figures 4B–D). KO-level PCoA showed a similar distribution (PC1 = 44.7%, PC2 = 13.3%), although group separation was less pronounced than at the EC level (Figures 4E–G). These findings reflect predicted metabolic potential based on taxonomic composition rather than directly measured functional activity.

#### 3.5.5 Functional pathways: KEGG and MetaCyc annotation

Predicted functional pathways were characterized using KEGG and MetaCyc annotations derived from PICRUSt2. At KEGG level 2 (Figures 4H,I), metabolism-related categories accounted for over 70% of predicted functions, with carbohydrate, amino acid, lipid, and energy metabolism predominating. Genetic Information Processing and Environmental Information Processing pathways were also represented, whereas Human Diseases-related pathways contributed minimally. Overall, functional profiles were consistent with taxonomic composition, highlighting metabolism-related processes as dominant features.

At MetaCyc level 3 (Figures 4J,K), biosynthetic pathways predominated, including fatty acid, lipid, amino acid, and carbohydrate biosynthesis. The “Generation of Precursor Metabolites and Energy” category was enriched for fermentation and glycolysis pathways. Degradation pathways were primarily associated with aromatic and chlorinated compounds. Frequency analysis aligned with relative abundance patterns, with fermentation and glycolysis pathways being the most prevalent, followed by degradation pathways. Detoxification and glycan-related pathways were less abundant but consistently detected across groups.

### 3.6 RT-qPCR validation of inflammatory markers

**FIGURE 5:**
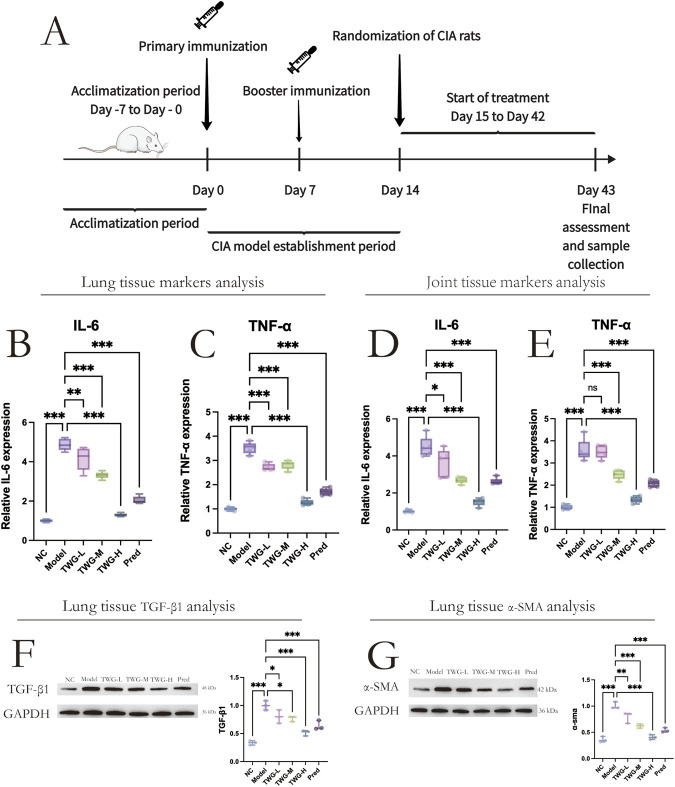
Molecular validation of the anti-inflammatory and anti-fibrotic effects of TWG. Groups: NC, normal control; model, CIA model; TWG-L, low-dose TWG; TWG-M, medium-dose TWG; TWG-H, high-dose TWG; Pred, prednisolone positive control. **(A)** Schematic diagram of the experimental design, grouping, and timeline of the CIA rat study. **(B,C)** Relative mRNA expression of IL-6 **(B)** and TNF-α **(C)** in lung tissues measured by RT-qPCR. **(D,E)** Relative mRNA expression of IL-6 **(D)** and TNF-α **(E)** in ankle joint tissues measured by RT-qPCR. **(F)** Representative WB bands and quantitative analysis of TGF-β1 protein expression in lung tissues. **(G)** Representative WB bands and quantitative analysis of α-SMA protein expression in lung tissues. For RT-qPCR analysis, five animals were randomly selected from each group. Panels **(B–E)** are shown as box plots. For WB analysis, three biological replicates were analyzed in each group, and the quantitative data in panels **(F,G)** are presented as mean ± SD. Statistical significance was determined using one-way ANOVA followed by Bonferroni’s multiple comparisons test. ns, not significant; *p < 0.05, **p < 0.01, ***p < 0.001 vs. model group.

RT-qPCR analysis was performed to evaluate the expression of IL-6 and TNF-α mRNA in lung and ankle joint tissues (Figures 5B–E).

In lung tissue, IL-6 mRNA expression was markedly elevated in the model group compared with the NC group (p < 0.001; Figure 5B). TWG treatment reduced IL-6 expression in a dose-dependent manner: TWG-L produced a modest but significant reduction (p < 0.05), whereas TWG-M and TWG-H induced more pronounced decreases (both p < 0.001). The TWG-H group achieved levels comparable to those observed with prednisolone. Similarly, pulmonary TNF-α mRNA expression was significantly increased in the model group (p < 0.001; Figure 5C). TWG-L did not significantly alter TNF-α expression compared with the model group (ns), whereas TWG-M and TWG-H significantly suppressed TNF-α mRNA (both p < 0.001), with the TWG-H group reaching expression levels similar to those of the prednisolone group (p < 0.001 vs. model).

In ankle joint tissue, IL-6 mRNA expression was also markedly elevated in the model group relative to NC (p < 0.001; Figure 5D). TWG treatment resulted in dose-dependent reductions, with TWG-L showing a significant decrease (p < 0.01) and TWG-M and TWG-H producing greater suppression (both p < 0.001). The TWG-H group exhibited the most pronounced attenuation, with expression levels approaching those of the NC group and comparable to prednisolone. Joint TNF-α mRNA expression was likewise significantly increased in the model group than in NC (p < 0.001; Figure 5E). All TWG doses significantly reduced joint TNF-α expression versus the model group (all p < 0.001), with a clear dose-dependent pattern; the prednisolone group likewise demonstrated significant suppression (p < 0.001 vs. model).

These findings indicate that TWG dose-dependently suppresses the transcriptional upregulation of key pro-inflammatory cytokines in both lung and joint tissues, consistent with the observed histopathological improvements.

### 3.7 WB validation of fibrotic markers

WB analysis was performed to assess TGF-β1 and α-SMA protein expression in lung tissue (Figures 5F,G). Compared with the NC group, the model group exhibited significantly elevated expression of both markers, indicating enhanced pulmonary fibrosis. TWG treatment reduced TGF-β1 and α-SMA expression to varying degrees. For TGF-β1, a significant reduction was observed only in the TWG-H group, whereas TWG-L, TWG-M, and Pred groups showed decreasing trends that did not reach statistical significance after Bonferroni correction. For α-SMA, significant reductions were observed in the TWG-M, TWG-H, and Pred groups, while the TWG-L group showed a non-significant downward trend. Among all treatment groups, TWG-H demonstrated the most pronounced inhibitory effect on both TGF-β1 and α-SMA expression, further supporting its role in attenuating pulmonary fibrotic changes in CIA rats.

## 4 Discussion

This study systematically evaluated the dose-dependent effects of TWG, a botanical drug-derived metabolite mixture, on joint injury, pulmonary interstitial lesions, and gut microbiota alterations in CIA rats. Overall, TWG alleviated both articular and pulmonary manifestations in a dose-dependent manner, with the TWG-H group showing the most pronounced therapeutic effects, comparable to prednisolone. These findings indicate that TWG exerts coordinated protective effects on joint and lung injury in this experimental model.

Histopathological findings further supported the overall therapeutic effects. In ankle joints, the model group exhibited marked synovial hyperplasia, inflammatory cell infiltration, and cartilage and bone destruction, whereas TWG treatment alleviated these pathological changes in a graded manner. In lung tissue, TWG improved alveolar septal thickening, inflammatory infiltration, and collagen deposition, particularly in the TWG-H group. These observations are consistent with previous reports demonstrating that TWG metabolites reduce arthritis severity, ameliorate tissue injury, and suppress inflammatory activity in experimental arthritis models (Tian et al., 2025; Zhu et al., 2022). Prior studies have also shown that TWG inhibits IL-8/CXCR2 signaling, suppresses NF-κB activation, and reduces the expression of osteoclast-related enzymes such as CTSK, TRAP, and MMP-9, thereby limiting joint destruction and inflammatory progression (Tian et al., 2025). In pulmonary tissues, TWG metabolites have been reported to inhibit myofibroblast differentiation, suppress excessive type I collagen production, and mitigate pathological remodeling in fibrotic disease models (Li et al., 2021; Yuan et al., 2019). Together, these findings support the view that TWG exerts multifaceted protective effects against both inflammatory and fibrotic tissue injury.

The newly added molecular validation experiments further strengthened these pathological observations. RT-qPCR analysis showed that TWG reduced IL-6 and TNF-α mRNA expression in both joint and lung tissues, although the inhibitory effect on lung TNF-α did not reach statistical significance in the TWG-L group. WB analysis further demonstrated that TWG reduced the expression of fibrosis-related proteins in lung tissue. TGF-β1 was significantly decreased in the TWG-H group, whereas α-SMA was significantly reduced in the TWG-M, TWG-H, and Pred groups. These results indicate that TWG not only improved histopathological injury but also suppressed key inflammatory cytokines and fibrotic markers, thereby providing direct molecular support for its coordinated anti-inflammatory and anti-fibrotic effects. This interpretation is consistent with previous studies showing that TWG reduces TNF-α, IL-6, IL-1β, and IL-17 in both *in vivo* and *in vitro* models (Li et al., 2021; Shan et al., 2023; Xu et al., 2025; Guo et al., 2025). Given that IL-6 and TNF-α are major therapeutic targets in RA, these findings are mechanistically consistent with the clinical and pathological improvements observed in the present study (Chen et al., 2025). In addition, recent evidence highlights the importance of the TGF-β1 axis in fibroblast activation and fibrotic remodeling, and butyrate has been shown to suppress TGF-β1-induced alveolar myofibroblast differentiation and the expression of fibrosis-related markers, supporting the biological relevance of the TGF-β1/α-SMA axis in the present study (Lee et al., 2021).

Gut microbiome analyses further suggest that the TWG dose is an important determinant of dysbiosis modulation. High-dose TWG was associated with increased α-diversity and a microbial community structure closer to that of the NC group, indicating partial restoration of intestinal ecological balance. In the TWG-H group, the relative abundance of *Lactobacillus* was comparable to that in the NC group, whereas *Bifidobacterium* and several putative butyrate-producing genera, including *Roseburia*, *Coprococcus*, *Oscillospira*, and *Ruminococcus*, were enriched. By contrast, taxa frequently associated with dysbiosis or pro-inflammatory states, such as *Desulfovibrio*, *Bilophila*, and *Prevotella*, were reduced following TWG treatment. These taxonomic shifts are biologically plausible, as disruption of gut microbiota and metabolite homeostasis has been increasingly linked to RA pathogenesis and immune dysregulation (Li and Kuhn, 2025; Xie et al., 2025). Notably, *Akkermansia* abundance was elevated in the model group and reduced across all treatment groups. Although *Akkermansia* is often considered beneficial, its effects are highly context-dependent (Cani and de Vos, 2017; Cani et al., 2022). Under physiological conditions, it may support barrier homeostasis, whereas under chronic inflammatory or dysbiotic conditions, excessive expansion of this mucin-degrading taxon may contribute to mucus layer thinning and increased susceptibility to inflammation (Tingler and Engevik, 2025). Therefore, its reduction following TWG treatment should be interpreted cautiously and may reflect a broader shift in mucosal ecology rather than a definitive improvement in barrier function. Direct assessments of intestinal barrier integrity and mucus-associated markers are required to clarify this relationship.

Beyond taxonomic compositional shifts, PICRUSt2-based functional inference suggested that the TWG-H group exhibited increased predicted metabolic potential for pathways related to SCFA metabolism. In parallel, TWG administration, particularly in the TWG-H group, increased the relative abundance of putative SCFA-producing genera, including *Lactobacillus*, *Ruminococcus*, and *Roseburia*. These findings are consistent with enhanced fermentation- and carbohydrate metabolism-related potential, although they do not directly demonstrate increased SCFA production. In RA, butyrate and other SCFAs have been reported to support immune homeostasis by enhancing IL-10-mediated B-cell function, inhibiting Th1/Th17 responses, and promoting Treg expansion (Rosser et al., 2020; Mizuno et al., 2017). SCFAs may also exert systemic immunomodulatory effects by strengthening epithelial barrier integrity, shaping T-cell differentiation, and regulating innate immune activation (Xie et al., 2025; Drakopanagiotakis et al., 2022; Zheng et al., 2023; Mikhail and O'Dwyer, 2025). Consistently, oral butyrate administration has been shown to alter lung macrophage subsets and attenuate pulmonary fibrosis in animal models (Drakopanagiotakis et al., 2022; Zhao et al., 2023). Recent studies further suggest that gut microbiota-derived metabolites may participate in lung fibrotic regulation through the gut-lung axis and influence TGF-β1-associated remodeling pathways (Zhou et al., 2025; Yang et al., 2025). In the present study, the enrichment of putative SCFA-producing genera and the PICRUSt2-predicted increase in SCFA-related metabolic potential are consistent with the hypothesis that TWG, particularly at higher doses, may promote a more anti-inflammatory metabolic milieu. At the same time, the newly added qPCR and WB results provide direct evidence that TWG suppresses IL-6, TNF-α, TGF-β1, and α-SMA in joint and lung tissues. Nevertheless, these data do not establish a causal link between microbial alterations and molecular improvement in the lung. Thus, the proposed gut-lung axis mechanism remains biologically plausible but not yet experimentally confirmed.

There are several limitations. First, the TWG used was a commercially available tablet preparation, and batch-level chemical characterization, such as LC-MS profiling, was not performed. Second, although RT-qPCR and WB analyses were included to validate selected inflammatory and fibrotic markers, only a limited number of targets were assessed, and the underlying mechanisms were not fully elucidated. Third, microbial functional conclusions were based on 16S rRNA sequencing combined with PICRUSt2 prediction, without validation by shotgun metagenomics, metabolomics, or quantitative measurement of key metabolites such as SCFAs. Fourth, microbiota-dependence experiments were not conducted, including antibiotic depletion, germ-free or pseudo-germ-free models, or fecal microbiota transplantation. Therefore, the requirement of gut microbiota for the therapeutic effects of TWG was not directly tested. Finally, immune-cell profiling was not performed, and further pharmacological and toxicological studies are needed to define the systemic toxicity, therapeutic window, optimal dosing regimen, and translational relevance of TWG. These limitations should be addressed in future studies to strengthen the mechanistic and translational interpretation of the present findings.

Future work should focus on: (Aletaha et al., 2010): integrating shotgun metagenomics with targeted and untargeted metabolomics, together with pathway-specific assays, to validate key functions inferred from the current 16S rRNA and PICRUSt2 analyses and to strengthen hypothesis testing beyond predictive profiling; (Wu et al., 2025); employing antibiotic pretreatment, germ-free or pseudo-germ-free models, fecal microbiota transplantation, and defined single-strain or microbial consortium interventions to determine whether the gut microbiota is required for TWG-associated benefits and to identify candidate taxa linked to these effects; and (Johnson et al., 2023) further evaluating the safety and tolerability of TWG in RA-ILD, while exploring the feasibility of combining TWG with microbiome-targeted interventions in preclinical models to support clinical translation as an integrated anti-inflammatory and antifibrotic therapeutic strategy for RA-ILD.

## Data Availability

The original contributions presented in the study are included in the article/Supplementary Material, further inquiries can be directed to the corresponding authors.

## Supplementary material

SUPPLEMENTARY FIGURE S1 Pairwise differential taxonomic analysis of gut microbiota based on LEfSe. **(A-O)** LEfSe (Linear Discriminant Analysis Effect Size) analysis showing significantly different taxa between pairwise group comparisons, including NC vs. TWG-L **(A)**, NC vs. TWG-H **(B)**, NC vs. TWG-M **(C)**, NC vs. model **(D-E)**, TWG-L vs. TWG-H **(F)**, TWG-L vs. TWG-M **(G)**, TWG-L vs. model **(H)**, TWG-L vs. Pred **(I)**, TWG-H vs. TWG-M **(J)**, TWG-H vs. model **(K)**, TWG-H vs. Pred **(L)**, TWG-M vs. model **(M)**, TWG-M vs. Pred **(N)**, and model vs. Pred **(O)**. Upper panels display LDA score bar plots, in which colored bars represent taxa significantly enriched in each group, and bar length indicates the effect size. Lower panels show corresponding LDA score distributions with significance thresholds. The results demonstrate pronounced microbial alterations induced by RA-ILD modeling and reveal that TWG intervention at different doses and prednisolone treatment modulate distinct taxonomic signatures.

SUPPLEMENTARY TABLE S1 Somatic and organ indices across groups.

SUPPLEMENTARY TABLE S2 Per-sample sequencing read statistics.

SUPPLEMENTARY TABLE S3 Numbers of ASVs and taxonomic units at different taxonomic ranks.

SUPPLEMENTARY TABLE S4 Relative abundance of dominant bacterial taxa across groups.

SUPPLEMENTARY TABLE S5 Diversity analysis of gut microbiota.
