# Supplementary figures and images for: Dose-dependent protective effects of Tripterygium wilfordii glycosides on joint and lung injury and gut microbiota remodeling in collagen-induced arthritis rats
Source: Front Pharmacol. 2026 Apr 23;17:1783503. doi: 10.3389/fphar.2026.1783503 (PMC13149293; doi:10.3389/fphar.2026.1783503)

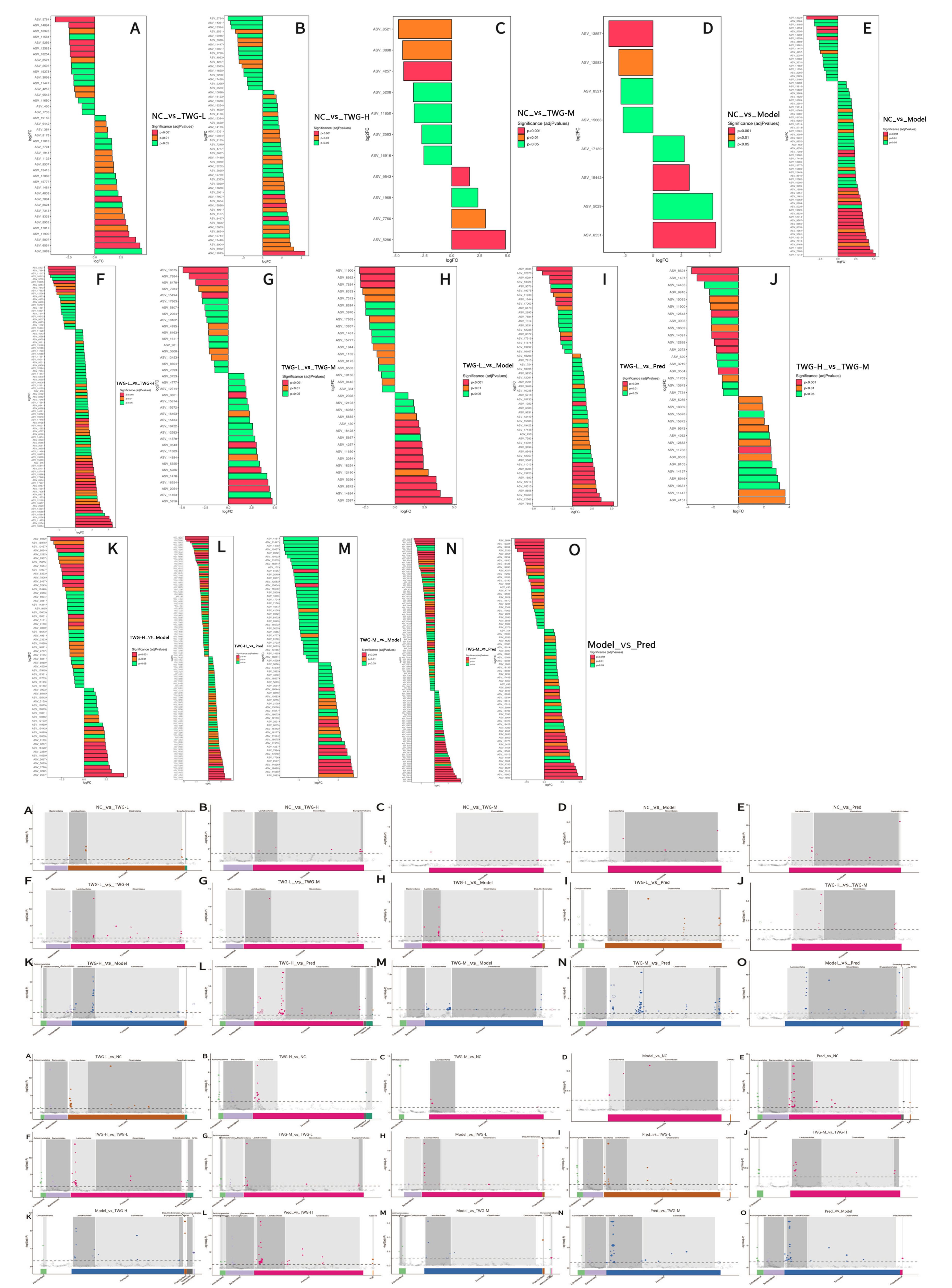

Supplement: Supplementary file 3 [file Image1.tif]
